# Coevolutionary patterns between coloration and diel activity in moths

**DOI:** 10.1098/rsos.250543

**Published:** 2025-07-09

**Authors:** Sohee Yoo, Yongsu Kim, Wonbin Lim, Dohyun Rim, Karl Loeffer-Henry, Thomas N. Sherratt, Changku Kang

**Affiliations:** ^1^ Department of Bioscience, Mokpo National University, Muan-gun, Jeollanam-do, South Korea; ^2^ Department of Agricultural Biotechnology, Seoul National University, Gwanak-gu, South Korea; ^3^ Department of Aerospace Engineering, Seoul National University, Gwanak-gu, South Korea; ^4^ Department of Biology, Carleton University, Ottawa, Ontario, Canada; ^5^ Research Institute of Agriculture and Life Sciences, Seoul National University, Gwanak-gu, South Korea

**Keywords:** anti-predator, diurnal, nocturnal, crepuscular, camouflage, aposematism

## Abstract

Anti-predator coloration is part of a key survival strategy in animals, often coevolving with behavioural traits such as diel activity. While previous studies have explored the link between conspicuous sexual signals and diurnality, the association between defensive coloration and diel activity remains unresolved. Here, we investigate the coevolutionary relationship between anti-predator coloration and diel activity in moths, a diverse clade with variable colour and activity patterns. Using a dataset of 322 moth species, we classified coloration of each species as cryptic or conspicuous and diel activity as diurnal, nocturnal, crepuscular or both day and night active (‘All’). We applied phylogenetic comparative methods to assess evolutionary transitions between these traits. Our findings suggest that moths ancestrally exhibited cryptic coloration and nocturnality. Conspicuous coloration was more frequent in diurnal species, supporting an evolutionary association between daytime activity and being conspicuous. Transitions between nocturnal and diurnal activity occurred predominantly through an intermediate All state, particularly in cryptic species. Cryptic diurnality also evolved in some lineages, possibly driven by specific ecological factors such as when thermoregulatory needs are high. These findings provide insights into the interplay between diel activity and anti-predator coloration, with implications for understanding correlated evolution between these two traits.

## 1. Introduction

Coloration in animals has long been recognized to play a role in reducing predation [1–3]. The mechanisms by which animal coloration protects prey have been extensively studied, with common forms of anti-predator coloration including camouflage and aposematism [4,5]. Camouflage is arguably the most widespread anti-predator strategy in nature and works by preventing detection or recognition of prey by predators [6,7]. In contrast, aposematism describes the combination of defensive traits, such as toxin, venoms or spines with conspicuous signals that advertise their unprofitability to predators [8]. While various sensory channels are used for both camouflage and aposematism [9–11], visual camouflage and aposematism have received the most attention from biologists.

Anti-predator coloration often coevolves with behavioural traits that enhance survival [12,13]. For example, threatening displays are commonly observed in species with bright coloration, sometimes only transiently exhibited during displays, which increase the likelihood that predators will abandon an attack [14,15]. Additionally, aposematism is often associated with bold behaviours, such as short escape initiation distances or active defence strategies [16–19]. In contrast, camouflaged species generally remain motionless, as movement can easily disrupt their concealment [20–22]. This fundamental difference may also influence, or be influenced by, a species’ diel activity. Movement is a real problem for camouflaged species: when a cryptic prey item moves, it is much more detectable [23]. If a species is under selective pressure to remain cryptic, natural selection may favour individuals that remain motionless during the day and restrict their activity to night-time, thereby reducing the risk of detection by visually oriented predators [24]. In contrast, aposematic species may not be constrained to a nocturnal lifestyle, as diurnal activity could provide additional benefits, such as enhanced resource collection [25,26]. Consequently, it has been hypothesized that diel activity patterns and defensive coloration are closely associated [27,28].

A few comparative studies have explored the coevolutionary patterns between coloration and diel activity. Emberts & Wiens [27] analysed the body coloration and diel activity of various terrestrial vertebrates and found that conspicuous sexual signals are associated with diurnality, whereas conspicuous warning coloration showed no clear association with diel activity type. More recently, Ribeiro *et al.* [28] demonstrated that diurnal dung beetle species are generally more conspicuous than non-diurnal ones, with male sexual dichromatism more commonly found in diurnal species. Collectively, these studies suggest that diel activity may drive the evolution of conspicuous sexual signals; however, evidence for an association between anti-predator coloration and diel activity remains either unsupported or inconclusive [22].

In this study, we examine the co-evolutionary patterns between diel activity and anti-predator coloration using moths as a model system. Moths provide a good group to test the association between diel activity and defensive coloration because both their colour patterns and diel activities are diverse, contrary to the common belief that all or at least most moths are nocturnal [29]. Furthermore, their colour patterns primarily serve an anti-predator function, while their role in sexual signalling has been scarcely reported [30], limiting the primary function of their coloration to predator deterrence.

Recently, Kawahara *et al.* [29] conducted an extensive literature review and compiled a diel activity dataset for Lepidoptera [29]. Importantly, instead of using the commonly applied dichotomous classification (diurnal versus nocturnal), they defined four categories: diurnal, nocturnal, active during both day and night and crepuscular. This allows us to conduct a more nuanced examination of the temporal niche of species that has been largely overlooked. Using modern phylogenetic analysis tools, we address the following key questions to explore the coevolutionary patterns between diel activity and coloration. First, is moth diel activity associated with specific types of anti-predator coloration? Consistent with observations from previous studies [27,28], we hypothesize that camouflaged appearances are more strongly associated with nocturnality than conspicuous appearances. Second, how have diel activity and anti-predator coloration evolved over time across the phylogeny? Specifically, we investigate: (i) whether transitions in anti-predator coloration have occurred in specific diel activity states, (ii) whether transitions in diel activity states have occurred in specific anti-predator coloration states, (iii) the contribution of largely overlooked diel activity states, such as being active during both day and night, to the dynamics of diel activity transitions and (iv) the sequence of anti-predator coloration and diel activity evolution from ancestral conditions.

## 2. Methods

### 2.1. Data acquisition and species colour classification

We obtained data on the diel activity of moths from Kawahara *et al.* [29]. They compiled diel activity data for both butterflies and moth species across almost all extant families by searching the best available resources and classified each species as diurnal (Diu), nocturnal (Noc), crepuscular (active during twilight hours at dawn and/or dusk) or all (active during both day and night). While the terms such as cathemerality (irregular activity intervals during both day and night) or arrhythmic (activity occurring both day and night) have been developed previously [30], we adopted the term ‘All’ from Kawahara *et al.* [29] (herein referred to as the All state) when a species was reported to be active during both day and night. This classification was retained because the All category by Kawahara *et al*.’s [29] accounts for any daytime and night-time activities that include sexually dimorphic diel activities, latitudinal variation within the same species and individual variations in a population, which are distinguished from cathemerality or arrhythmic. Butterflies (superfamily Papilionoidea) in this dataset were predominantly diurnal, leaving insufficient variation in diel activity for comparative analyses. Thus, we excluded butterflies and retained only moths in our analysis.

To classify whether each species exhibits a cryptic (Cry) or conspicuous (Con) appearance, we gathered images of each species from various online image databases (electronic supplementary material, table S3). We collected three to five images per species, including both natural and specimen photographs whenever possible. Once all images were obtained, we conducted expert classifications: four experts in colour research (T.N.S., K.L.-H., C.K. and Y.K.) independently classified each species as cryptic, conspicuous or uncertain after observing the collected images. Classification was based on the overall morphology of the resting posture following previous criteria used in moths [31]. We classified a species as Cry if the colour patterns of both its forewings and hindwings resembled natural substrates such as leaves, tree bark, dead leaves or lichens (e.g. shades of green, brown or grey). A species was considered Con if its forewings displayed colours typically associated with aposematism (e.g. orange, yellow, red, purple, blue or white), often with highly contrasting patterns, regardless of hindwing coloration.

For species with internal contrasting patterns on their forewings, we carefully distinguished between disruptive contrast, which evolved for camouflage, and aposematic contrast [32]. Disruptive contrast typically included at least one colour resembling natural substrates, whereas aposematic contrast featured colours commonly linked to warning signals. Some moth species (11 species) exhibit cryptic resting colours on forewings with a conspicuous colour hidden usually on hindwings, presumably evolved for deimatism or flash behaviour [33–35]. Rather than creating a separate classification group, we classified these hidden-coloured species as Cry because: (i) they typically adopt a camouflaged appearance when at rest, (ii) the number of hidden-coloured species in our dataset was limited, which makes it difficult to analyse these species as a separate group and (iii) no specific predictions exist regarding the association between hindwing coloration and diel activity. When a species exhibited polymorphism, we classified both forms. If both forms fell into the same colour group, we assigned the species to that group. Otherwise, we excluded the species from the analysis due to uncertainty regarding its defensive coloration.

The Kawahara *et al.* [29] dataset consisted of 449 moth species with known diel activity. Among them, we obtained images of 363 species. After the independent classifications, we compiled a dataset including only species for which at least three of the four experts agreed on the classification; this dataset, consisting of 322 species, was used as the main dataset. One species classified as uncertain was excluded. Additionally, we generated a second dataset (*n* = 239) consisting only of species for which all four experts agreed on the classification and compared whether the results remained consistent.

### 2.2. Time-calibration of phylogenetic tree and phylogenetic analysis

All phylogenetic analyses were conducted in the R environment [36]. First, we time-calibrated the tree by Kawahara *et al.* [29] to generate an ultrametric chronogram using the ‘chronos’ function in the ‘ape’ package [37]. We used 13 calibration points, carefully selected in a previous systematic study [38] (electronic supplementary material, figure S1). The minimum and maximum ages of each node were inferred from TimeTree (timetree.org). The ‘chronos’ function allowed us to assume either ‘relaxed’, ‘correlated’, ‘discrete’ or ‘strict clock’ substitution models [39]. The calibrated branch lengths differ only subtly across the models except for the strict clock model (electronic supplementary material, figure S2). Nonetheless, we performed phylogenetic analyses on all four trees and compared the results. We primarily report the results based on the correlated model tree and present the results from the other trees in the electronic supplementary material.

We first generated various hypothetical models to fit our data to investigate the transitional patterns between different colour and diel activity states. Our dataset included eight different states (two colour states × four diel activity states). However, species in the crepuscular state were rare and predominantly classified as cryptic, making it difficult to incorporate this group into hypothesis generation because the estimated transition rates are often unreliable when the number of species in a category is too small. Therefore, we excluded the crepuscular state and used only six states (two colour states × three diel activity states) when constructing hypothetical models. The generation of different models was done by constraining the rates of specific transitional paths to zero.

**Figure 1.**
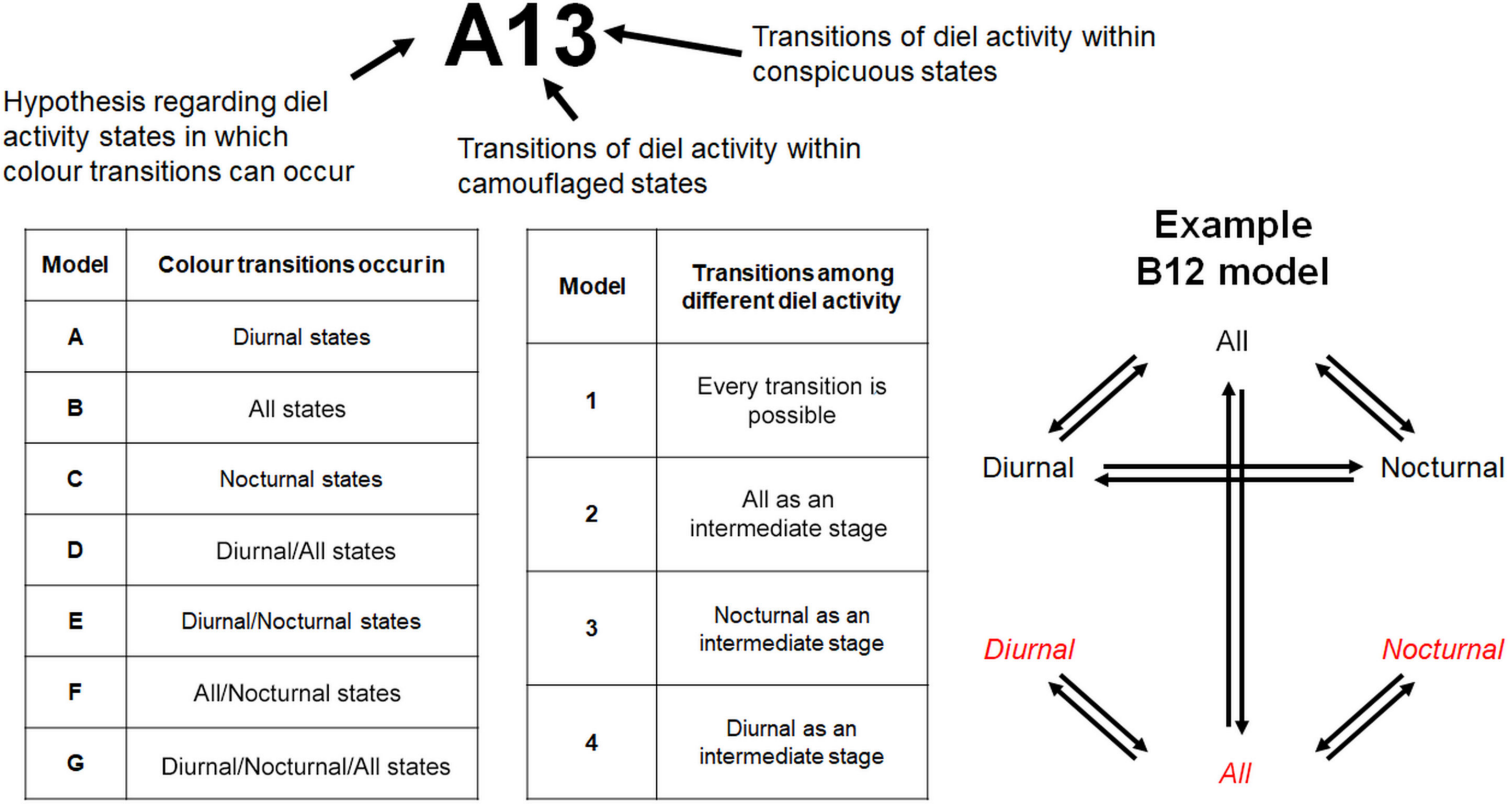
The name and structure of the hypothetical models. Text in black refers to transitions in diel activity within camouflaged states, while text in red refers to transitions in diel activity within conspicuous traits.

We developed hypothetical models encompassing all possible transitional scenarios. We did not allow simultaneous changes in colour and diel activity (dual transitions) as simultaneous changes in two independent traits are expected to be less probable than changes in a single trait. While this assumption is not based on direct empirical evidence, it reflects a common modelling approach when transitions in multiple traits are considered independently evolving. We used alphabetic labels to denote models that describe (i) the diel activity states at which transitions between cryptic and conspicuous coloration occurred, followed by two numeric digits indicating (ii) the nature of transitions between different diel activity states within the cryptic and conspicuous colour states (figure 1). This resulted in 112 (7 × 4 × 4) candidate models, named from A11 to G44. All hypothetical models assume that the estimated transition rates differ between each path. We also assumed no irreversible transitions to keep the number of hypotheses manageable. However, any irreversible transitions would still be detectable from the estimated transition rates.

**Figure 2.**
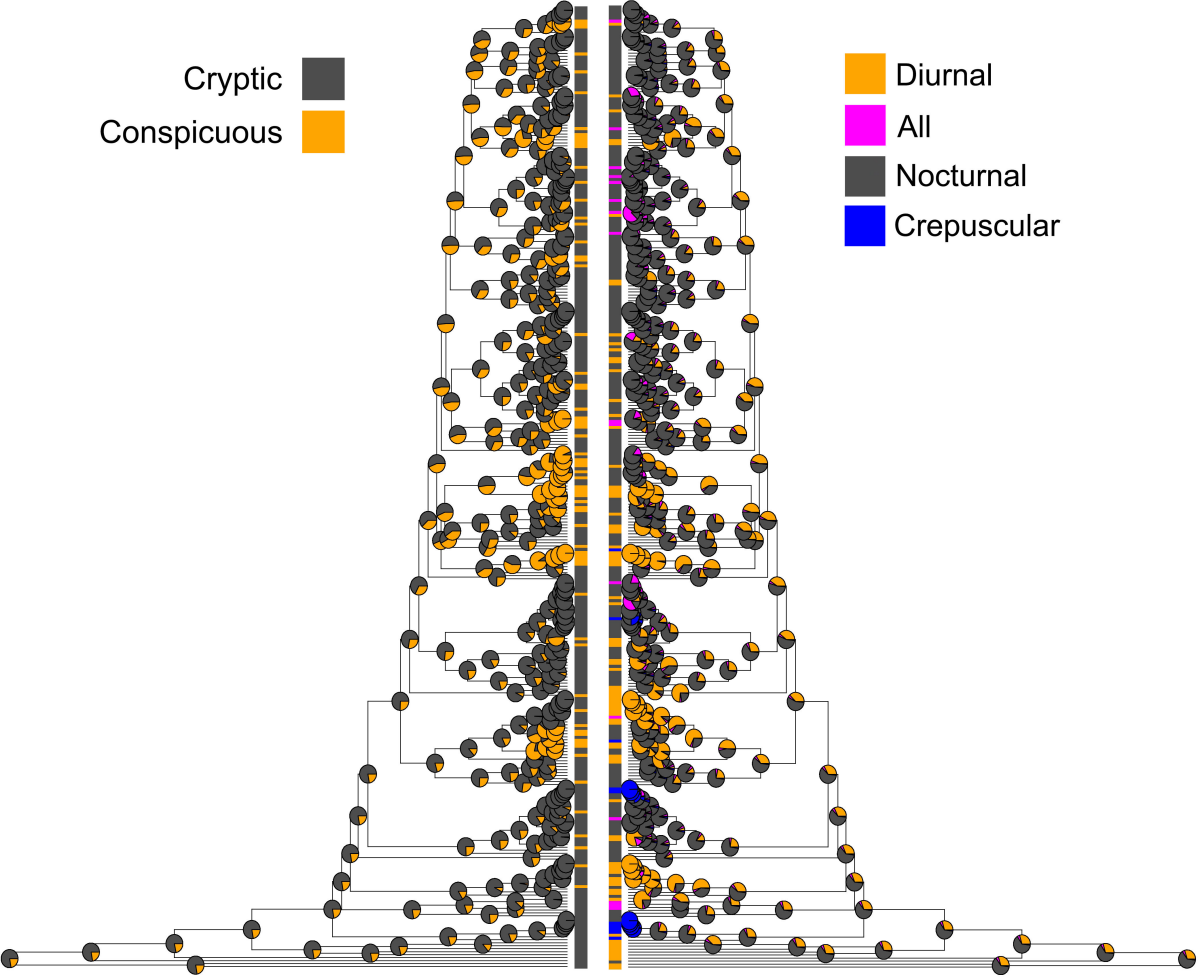
The ancestral character reconstruction for coloration (left) and diel activity (right). The pie charts at each node represent the marginal probabilities of the ancestral states.

Although we did not include crepuscular state into hypothetical model generation, we included crepuscular species as a separate state in the analysis and estimated the transitions from or to crepuscular state to all other diel activity states to account for any contributions of crepuscular states to transition dynamics, while refraining from testing specific hypotheses regarding crepuscular states. We consider the contribution of crepuscular states to past transition dynamics to be minor, not only because the number of species is low, but also because most crepuscular states evolved recently near the tree tip (see figure 2), limiting their contributions to transition dynamics to only closely related species, if any.

We then fitted extended Mk (M for Markov and k for number of discrete states) models using the ‘fitMk’ function in the ‘phytools’ package to estimate transition rates between each state according to each hypothesis [40,41]. These rates represent the probabilities of transitioning from one state to another under a Markov process. Once we obtained results from all models, we assessed model fit using Akaike information criterion (AIC) and AIC weights.

Initially, we attempted information-theoretic model averaging to estimate model-averaged transition rates. However, this approach raised issues when making inferences from the averaged values, as some models produced excessively large estimates (>100, whereas typical rates are <1), disproportionately influencing the averages. Instead, we present a subset of the best-supported results that were consistent across all phylogenetic trees used. Based on these best-supported models, we also performed stochastic character mapping to estimate the number of state transitions in the phylogenetic tree for each path using the ‘simmap’ function in the ‘phytools’ package. We note that the transition rates estimated from a discrete character evolution model do not necessarily correlate with the number of past transitions inferred by stochastic character mapping. For example, the estimated transition rates could be high even if only a single transition event occurred, provided that this transition happened rapidly. Additionally, we inferred ancestral states and visualized the ancestral states of colour and diel activity at each node using the ‘corHMM’ function in the ‘corHMM’ package [42].

To examine whether the frequency of each type of defensive coloration differed between diel activity types, we fitted a phylogenetic generalized linear model using the ‘phyloglm’ function in the ‘phylolm’ package [43]. Defensive coloration type was used as the response variable and diel activity type as the predictor. *Post hoc* comparisons were conducted using custom R code available on Figshare [44]. Values of *p* were adjusted to control for false discovery rate [45].

## 3. Results

**Figure 3.**
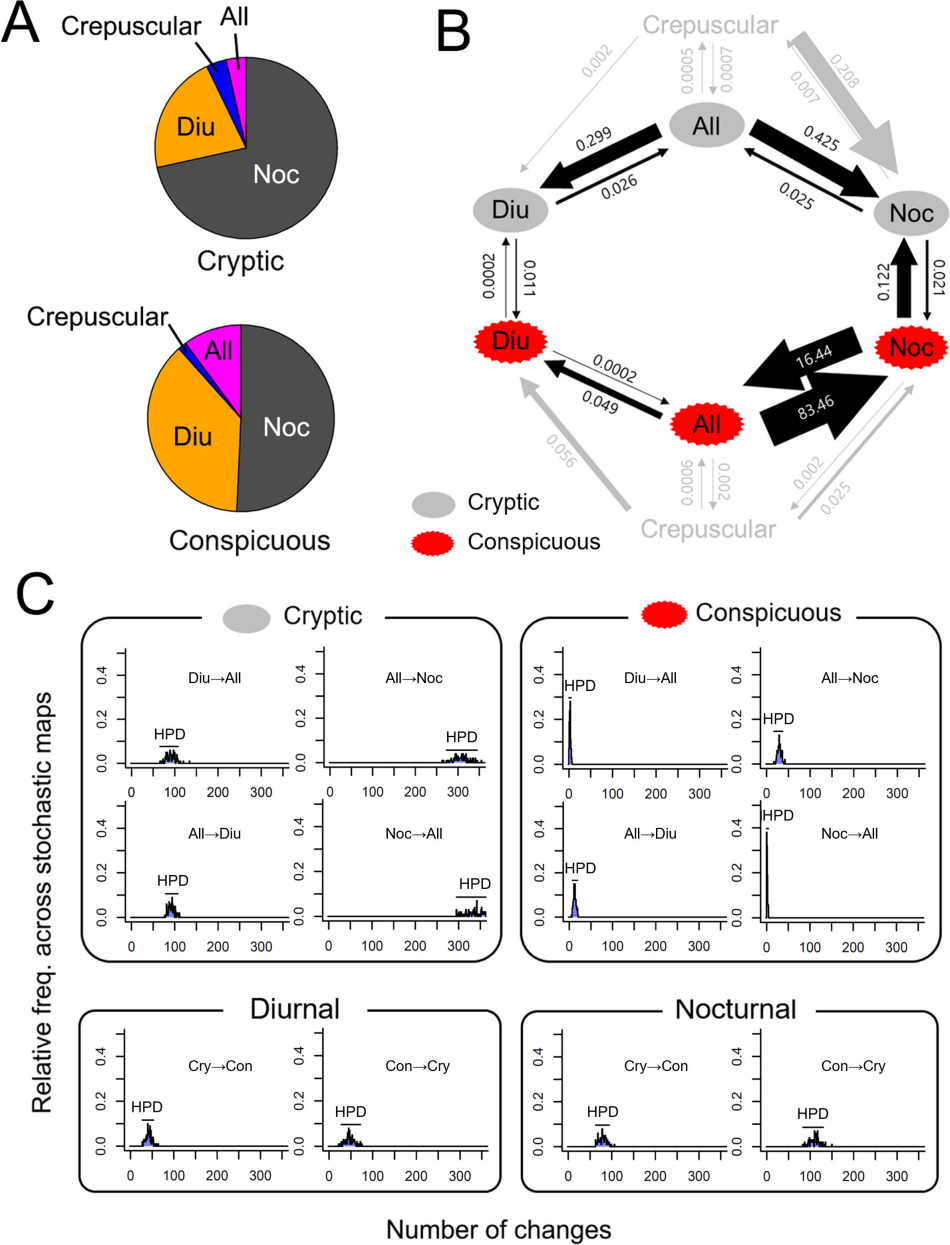
The proportion of species with each diel activity state for each defensive colour type (A), and the results of best-supported model (E22) from discrete character evolution model (B) and stochastic character mapping (C). Crepuscular states were excluded from the generation of hypothetical models due to the limited number of species exhibiting crepuscular activity. Nevertheless, transitions to and from crepuscular states were incorporated when estimating transition rates; however, they are depicted in light grey to reflect the low credibility of these estimates.

All four classifiers agreed for 239 species (65.8% among all species) and three classifiers agreed on 322 species (88.7%). The sample species images for each category are provided in electronic supplementary material, figure S3. The main analyses were conducted on the 322 species. Diurnal species were more likely to be conspicuous than nocturnal species (estimate = 0.83, s.e. = 0.32, *z* = 2.61 and *P*_adj_ = 0.05). We found no differences in defensive colour types among the other diel activity categories (all *P*_adj_ > 0.31). The proportion and frequency of each diel activity category within each colour type are depicted in figure 3A and electronic supplementary material, table S1.

When fitting extended Mk models, we encountered errors with some models (A22, A23, B33, B44 and C22) which is likely because they assume improbable transitional pathways given our data. Indeed, all models starting with the letters A, B and C with transitions from single states alone have very low AIC weight values (electronic supplementary material, table S2), which demonstrates that the omission of these non-fitted models would not affect the main results. Discrete character evolution models suggest that either E22 or E23 models (with colour transitions from diurnal and nocturnal states; see figure 1 for model explanations) were best supported or second-best supported across all trees, and the best model depended on the tree type (table 1). The estimated transition rates were similar across all trees except for the E22 model from the strict clock tree, where the transition rates of diel activity within conspicuously coloured species were different from the other three trees (electronic supplementary material, figure S4). We mainly show the results from correlated model tree, which qualitatively show the same results from the relaxed or discrete model trees.

**Table 1.** AIC and AIC weight values of the best five models for the phylogenetic trees that used different substitution models. Bold text shows the two best-supported models.

substitution rate model	transition model	logLik	d.f.	AIC	AIC weight
correlated	**E22**	**−397.76**	**24**	**843.51**	**0.21**
	**E23**	**−398.15**	**24**	**844.3**	**0.14**
	D22	−398.45	24	844.9	0.11
	E33	−398.82	24	845.64	0.07
	G32	−396.84	26	845.69	0.07
relaxed	**E23**	**−398.36**	**24**	**844.72**	**0.29**
	**E22**	**−398.48**	**24**	**844.96**	**0.26**
	D22	−399.33	24	846.66	0.11
	D21	−398.29	26	848.58	0.04
	E33	−400.34	24	848.68	0.04
discrete	**E23**	**−398.8**	**24**	**845.6**	**0.22**
	**E22**	**−398.86**	**24**	**845.71**	**0.2**
	D22	−399.31	24	846.61	0.13
	E33	−399.84	24	847.68	0.08
	G21	−396.33	28	848.66	0.05
strict clock	**E22**	**−378.29**	**24**	**804.58**	**0.16**
	**E23**	**−378.31**	**24**	**804.62**	**0.15**
	E13	−376.63	26	805.27	0.11
	E12	−376.65	26	805.29	0.11
	E42	−379.27	24	806.54	0.06

There exist several major patterns that are congruent across both E22 and E23 models regardless of the tree used. First, the most likely ancestral state of moths was the cryptic nocturnal (Cry/Noc) state (figure 2). Second, while the number of species is substantially less in All states than both Diu and Noc states, the All states served as an intermediate stage for the transition between Diu and Noc states in cryptically coloured moths. Within Con states, two diel activity transition scenarios were similarly supported: (i) All states served as an intermediate stage for the transition between Diu and Noc (E22 model) and (ii) Noc states evolved to either Diu or All states but no transitions occurred between Diu and All (E23 model). Third, the transition between Cry and Con states occurred either in Diu or in Noc states but not in All states. While the transitions can occur in both directions, Con to Cry transition rates were higher than the opposite direction in both Noc and Diu states. Fourth, All states are labile (i.e. the transition rates away from All states were greater than the transitions towards All states) and subsequently become either Diu or Noc states in both Cry and Con colours. Fifth, all non-nocturnal (i.e. non-ancestral) diel activity states evolved multiple times independently (figure 2).

The results of stochastic character mapping largely mirrored the transition rates results (figure 3C) with some notable exceptions: while the transition rates were estimated to be high, the frequency of the transitions between conspicuous nocturnal (Con/Noc) and conspicuous All (Con/All) rarely occurred. This suggests that the transition rates were inferred from a small number of rapid transitions between these states.

## 4. Discussion

The support for the E22 and E23 models suggests that (i) colour transitions occur in both nocturnal and diurnal states (alphabet ‘E’ models) and (ii) transitions between nocturnality and diurnality occur through intermediary All states, at least within cryptic states and possibly also within conspicuous states (number ‘2’ models). This corroborates the findings by Slavenko *et al.* [46] where transition between diurnality and nocturnality in skinks occurred in a stepwise manner through a cathemeral state. In particular, this pattern was robust in transitions within cryptically coloured moths. However, as the low number of species with both day and night activities suggests, this all-day active state is unstable and quickly transitions to either diurnal or nocturnal states. This pattern is less evident among conspicuously coloured species, although the all-day active state is similarly unstable, with a stronger tendency to revert to a nocturnal state. This might be due to the fact that the transitions within conspicuously coloured species occurred less frequently; indeed, stochastic character mapping showed that the frequency of the transitions of diel activities within conspicuously coloured species was substantially lower than the other transitional pathways (figure 3C).

Our results also suggest the evolutionary association between diel activity and coloration; just as Ribeiro *et al.* [28] reported in their analysis of dung beetles [28], diurnality was more frequently found in more colourful species. Multiple pathways could lead to the transition from an ancestral cryptic/nocturnal state to a conspicuous/diurnal state, the two states in which most species are found. This transition could occur via both cryptic diurnal and conspicuous All (or conspicuous nocturnal in E23 models) states. Our analysis also makes a single transitional pathway leading to the evolution of conspicuous/diurnal state highly unlikely. Stochastic character mapping results show similar patterns; while E22 models show that there have been relatively fewer transition events from conspicuous nocturnal to conspicuous All states, E23 model results demonstrate that the transitions from both cryptic diurnal and conspicuous nocturnal to conspicuous diurnal have occurred in similar frequencies (see figure 3C).

As described above, nocturnality is more frequently associated with cryptic rather than conspicuous appearance. This is consistent with the observation that movement during daytime breaks camouflage which would limit the evolution of diurnality in camouflaged species [23]. Still, one notable finding is that there exists a considerable number of species with the combination of cryptic coloration and diurnal activity (65 species in our data, comprising approx. 18% of the total). Diurnal cryptic species may evolve when the benefits of being active during the day, such as improved foraging, mate searching or thermoregulation, outweigh the associated costs. Kawahara *et al.* [29] suggested that many diurnal species that are nested in nocturnal clades are found at high latitudes or elevations or active during autumn and winter [29]. These geographical locations or seasons may either give thermoregulatory benefits to diurnal individuals or alleviate predatory burdens of being active during the day (or both), which could drive the evolution of diurnal activities in cryptic moths [47]. Cryptic diurnal species in our list are from diverse families, and their ecology is mostly unknown. However, there was a trend suggesting that species in small-sized micro-lepidopteran species are more likely to be diurnal than those in macro-lepidopteran species (electronic supplementary material, figure S5). Among species with diurnal or All diel activity, 86% of micro-lepidopterans are considered cryptic (*N* = 64), compared with 25% in macro-lepidopterans (*N* = 32). Possibly, the cost of being diurnal is lower for micro-lepidopteran species because their small size either (or both) prevents detection by predators, even during movement, or renders them less attractive as prey [48,49].

Although our analysis includes currently the best available diel activity data of moths, our sampling includes one or only a few species per genus which makes our analysis likely represent genus-level (or higher) transitional patterns while possibly missing species-level transitional patterns. While phylogenetic signal (the degree to which closely related species tend to share similar traits) of diel activities in lepidoptera is unknown, family-level diel activities are generally conserved with minor exceptional species [29], and phylogenetic signal is reported to be strong in other animal groups [50–52]. Thus, we consider that our results likely represent transitional patterns in higher level phylogenetic relationships but may miss transitional patterns that are present in recently diverged species. Also, our analyses are subject to topological uncertainties inherited from Kawahara *et al.* [29], which may influence phylogenetic inferences. Future studies would benefit from a more fully resolved tree that reduces these uncertainties.

While we did not seek to make inferences about the evolution of crepuscular activity, this state seems to be unstable and evolved only in certain lineages and perhaps in specific ecological circumstances. Odell & Mastro [53] argued that crepuscular activity of gypsy moth, *Lymantria dispar*, appears to be for reducing predation and helping males locate females. Some crepuscular moths are pollinators, but the adaptive benefits of being crepuscular are unknown [54]. Perhaps being crepuscular enables moths to reduce the encounters with predators or provides a foraging advantage when searching for nectar-rich flowers.

Naturally, our classification of moth coloration based on human perception has some limitations, but we expect the consequences to be minimal. An animal’s conspicuousness depends not only on its colour but also on the observer’s visual system, the background and the animal’s behaviour [55]. Natural predators such as birds have different visual sensitivities from humans. Although we did not account for predator vision, human assessments have been shown to provide useful insights in large comparative studies and correlate well with classifications based on bird vision [56–59]. Colours we scored as conspicuous (e.g. red, yellow and orange) likely generate strong chromatic and achromatic contrasts against most commonly found natural substrates, making them visible to common avian predators. Our reliance on web images also overlooks UV colours, but since natural backgrounds seldom reflect UV [60], UV coloration, if present, would generally appear conspicuous. Furthermore, UV colours in moths are typically associated with already conspicuous colours such as yellow, white, and blue [31,61], rather than cryptic patterns. As UV signals alone are unlikely to evolve due to the diversity of moth predators’ visual systems and the potential cost to camouflage, we expect the rate of misclassification due to ignoring UV to be low.

In conclusion, accounting for the underappreciated diel activity types (active both day and night, and crepuscular), our results provide evidence in moths that (i) conspicuous species are more likely to be diurnal than cryptic species, (ii) the shift between nocturnality and diurnality has been often mediated by an intermediate state involving activity across both day and night and (iii) there exist multiple pathways leading to conspicuous diurnal traits rather than a single route. The seemingly gradual changes in diel activity patterns are not surprising, as diel activities are polygenic traits, making abrupt changes unlikely, unlike qualitative traits [62,63]. The observed historical transitions of colour and diel activity in moths should be affected by the shift in ecological or physiological conditions, such as predation, thermoregulatory pressures, nectar availability or nocturnal colour vision [64], which remains an open area for future research.

## Data Availability

All data and analysis codes have been deposited at Figshare [44]. Electronic supplementary material is available online [65].

## References

[1] Cott HB. 1940 Adaptive coloration in animals. London, UK: Methuen.

[2] Wiens JJ, Emberts Z. 2025 How life became colourful: colour vision, aposematism, sexual selection, flowers, and fruits. Biol. Rev. **100**, 308–326. (10.1111/brv.13141) 39279365

[3] Caro T, Koneru M. 2021 Towards an ecology of protective coloration. Biol. Rev. **96**, 611–641. (10.1111/brv.12670) 33258554

[4] Stevens M, Merilaita S. 2011 Animal camouflage: mechanisms and function, pp. 1–16. New York, NY: Cambridge University Press. (10.1017/CBO9780511852053.001)

[5] Ruxton GD, Allen WL, Sherratt TN, Speed MP. 2018 Avoiding attack: the evolutionary ecology of crypsis, aposematism, and mimicry, 2nd edn, pp. 84–102. Oxford, UK: Oxford University Press. (10.1093/oso/9780199688678.001.0001)

[6] Skelhorn J, Rowland HM, Speed MP, Ruxton GD. 2010 Masquerade: camouflage without crypsis. Science **327**, 51. (10.1126/science.1181931) 20044568

[7] Stevens M, Merilaita S. 2009 Animal camouflage: current issues and new perspectives. Phil. Trans. R. Soc. B **364**, 423–427. (10.1098/rstb.2008.0217) 18990674 PMC2674078

[8] Poulton EB. 1890 The colours of animals: their meaning and use, especially considered in the case of insects. London, UK: D. Appleton. (10.5962/bhl.title.30570)

[9] Ruxton GD. 2009 Non-visual crypsis: a review of the empirical evidence for camouflage to senses other than vision. Phil. Trans. R. Soc. B **364**, 549–557. (10.1098/rstb.2008.0228) 19000976 PMC2674081

[10] Weldon PJ. 2013 Chemical aposematism. Chemoecology **23**, 201–202. (10.1007/s00049-013-0140-3)

[11] Hristov NI, Conner WE. 2005 Sound strategy: acoustic aposematism in the bat–tiger moth arms race. Naturwissenschaften **92**, 164–169. (10.1007/s00114-005-0611-7) 15772807

[12] Brodie ED III. 1992 Correlational selection for color pattern and antipredator behavior in the garter snake Thamnophis ordinoides. Evolution **46**, 1284–1298. (10.1111/j.1558-5646.1992.tb01124.x) 28568995

[13] Forsman A, Appelqvist S. 1998 Visual predators impose correlational selection on prey color pattern and behavior. Behav. Ecol. **9**, 409–413. (10.1093/beheco/9.4.409)

[14] Hernández-Palma TL, Rueda-Solano LA, Valkonen JK, Rojas B. 2023 Predator response to the coloured eyespots and defensive posture of Colombian four-eyed frogs. J. Evol. Biol. **36**, 1040–1049. (10.1111/jeb.14193) 37341128

[15] Olofsson M, Løvlie H, Tibblin J, Jakobsson S, Wiklund C. 2013 Eyespot display in the peacock butterfly triggers antipredator behaviors in naive adult fowl. Behav. Ecol. **24**, 305–310. (10.1093/beheco/ars167) 23243378 PMC3518204

[16] Kojima W. 2022 Fearless distasteful butterflies and timid mimetic butterflies: comparison of flight initiation distances in Papilioninae. Biol. Lett. **18**, 20220145. (10.1098/rsbl.2022.0145) 35538843 PMC9091839

[17] Dowdy NJ, Conner WE. 2019 Nonchalant flight in tiger moths (Erebidae: Arctiinae) is correlated with unpalatability. Front. Ecol. Evol. **7**, 480. (10.3389/fevo.2019.00480)

[18] Blanchette A, Becza N, Saporito RA. 2017 Escape behaviour of aposematic (Oophaga pumilio) and cryptic (Craugastor sp.) frogs in response to simulated predator approach. J. Trop. Ecol. **33**, 165–169. (10.1017/S0266467417000037)

[19] Lartviere S, Messier F. 1996 Aposematic behaviour in the striped skunk, Mephitis mephitis. Ethology **102**, 986–992. (10.1111/j.1439-)

[20] Regan D, Beverley K. 1984 Figure–ground segregation by motion contrast and by luminance contrast. J. Opt. Soc. Am. A. **1**, 433. (10.1364/JOSAA.1.000433) 6726491

[21] Yin J, Gong H, An X, Chen Z, Lu Y, Andolina IM, McLoughlin N, Wang W. 2015 Breaking cover: neural responses to slow and fast camouflage-breaking motion. Proc. R. Soc. B **282**, 20151182. (10.1098/rspb.2015.1182) PMC4632627 26269500

[22] Merilaita S, Tullberg BS. 2005 Constrained camouflage facilitates the evolution of conspicuous warning coloration. Evolution **59**, 38–45. (10.1111/j.0014-3820.2005.tb00892.x) 15792225

[23] Hall JR, Cuthill IC, Baddeley R, Shohet AJ, Scott-Samuel NE. 2013 Camouflage, detection and identification of moving targets. Proc. R. Soc. B **280**, 20130064. (10.1098/rspb.2013.0064) PMC3619462 23486439

[24] Allen WL, Moreno N, Gamble T, Chiari Y. 2020 Ecological, behavioral, and phylogenetic influences on the evolution of dorsal color pattern in geckos. Evolution **74**, 1033–1047. (10.1111/evo.13915) 31886521

[25] Speed MP, Brockhurst MA, Ruxton GD. 2010 The dual benefits of aposematism: predator avoidance and enhanced resource collection. Evolution **64**, 1622–1633. (10.1111/j.1558-5646.2009.00931.x) 20050915

[26] Higginson AD, Speed MP, Ruxton GD. 2015 Florivory as an opportunity benefit of aposematism. Am. Nat. **186**, 728–741. (10.1086/683463) 26655980

[27] Emberts Z, Wiens JJ. 2022 Why are animals conspicuously colored? Evolution of sexual versus warning signals in land vertebrates. Evolution **76**, 2879–2892. (10.1111/evo.14636) 36221224

[28] Ribeiro PHO, Frizzas MR, Vaz-de-Mello FZ, Gawryszewski FM. 2024 The evolution of body coloration in dung beetles: diel activity and sexual dimorphism. Evol. Ecol. **38**, 449–460. (10.1007/s10682-024-10300-9)

[29] Kawahara AY, Plotkin D, Hamilton CA, Gough H, St Laurent R, Owens HL, Homziak NT, Barber JR. 2018 Diel behavior in moths and butterflies: a synthesis of data illuminates the evolution of temporal activity. Org. Divers. Evol. **18**, 13–27. (10.1007/s13127-017-0350-6)

[30] Cox DTC, Gaston KJ. 2024 Cathemerality: a key temporal niche. Biol. Rev. **99**, 329–347. (10.1111/brv.13024) 37839797

[31] Kim Y, Hwang Y, Bae S, Sherratt TN, An J, Choi SW, Miller JC, Kang C. 2020 Prey with hidden colour defences benefit from their similarity to aposematic signals. Proc. R. Soc. B **287**, 20201894. (10.1098/rspb.2020.1894) PMC7542798 32900312

[32] Cuthill IC, Székely A. 2009 Coincident disruptive coloration. Phil. Trans. R. Soc. B **364**, 489–496. (10.1098/rstb.2008.0266) 18990668 PMC2674087

[33] Umbers KDL, De Bona S, White TE, Lehtonen J, Mappes J, Endler JA. 2017 Deimatism: a neglected component of antipredator defence. Biol. Lett. **13**, 20160936. (10.1098/rsbl.2016.0936) 28404819 PMC5414691

[34] No S, Yang HM, Sherratt TN, Kang C. 2024 Flash behaviors protect prey from avian predators. Behav. Ecol. **35**, arae076. (10.1093/beheco/arae076)

[35] Kang C, Zahiri R, Sherratt TN. 2017 Body size affects the evolution of hidden colour signals in moths. Proc. R. Soc. B **284**, 20171287. (10.1098/rspb.2017.1287) PMC5577493 28855366

[36] R Core Team. 2024 R: a language and environment for statistical computing. Vienna, Austria: R Foundation for Statistical Computing. See https://www.R-project.org/.

[37] Paradis E, Claude J, Strimmer K. 2004 APE: analyses of phylogenetics and evolution in R language. Bioinformatics **20**, 289–290. (10.1093/bioinformatics/btg412) 14734327

[38] Kawahara AY *et al*. 2019 Phylogenomics reveals the evolutionary timing and pattern of butterflies and moths. Proc. Natl Acad. Sci. USA **116**, 22657–22663. (10.1073/pnas.1907847116) 31636187 PMC6842621

[39] Paradis E. 2013 Molecular dating of phylogenies by likelihood methods: a comparison of models and a new information criterion. Mol. Phylogenet. Evol. **67**, 436–444. (10.1016/j.ympev.2013.02.008) 23454091

[40] Revell LJ. 2024 phytools 2.0: an updated R ecosystem for phylogenetic comparative methods (and other things). PeerJ **12**, e16505. (10.7717/peerj.16505) 38192598 PMC10773453

[41] Lewis PO. 2001 A likelihood approach to estimating phylogeny from discrete morphological character data. Syst. Biol. **50**, 913–925. (10.1080/106351501753462876) 12116640

[42] Beaulieu J, O’Meara B, Oliver J, Boyko J. 2021 corHMM: hidden Markov models of character evolution. R package version 2.1. See https://CRAN.R-project.org/package=corHMM.

[43] Ho LST, Ané C. 2014 A linear-time algorithm for Gaussian and non-Gaussian trait evolution models. Syst. Biol. **63**, 397–408. (10.1093/sysbio/syu005) 24500037

[44] Yoo S, Kim Y, Lim W, Rim D, Loeffler-Henry K, Sherratt TN, Kang C. 2025 Data and R code for the paper ‘Coevolutionary patterns between coloration and diel activity in moths’. Figshare. Dataset. (10.6084/m9.figshare.28615955) PMC12308327 40740705

[45] Benjamini Y, Hochberg Y. 1995 Controlling the false discovery rate: a practical and powerful approach to multiple testing. J. R. Stat. Soc. **57**, 289–300. (10.1111/j.2517-6161.1995.tb02031.x)

[46] Slavenko A, Dror L, Camaiti M, Farquhar JE, Shea GM, Chapple DG, Meiri S. 2022 Evolution of diel activity patterns in skinks (Squamata: Scincidae), the world’s second-largest family of terrestrial vertebrates. Evolution **76**, 1195–1208. (10.1111/evo.14482) 35355258 PMC9322454

[47] van der Vinne V, Gorter JA, Riede SJ, Hut RA. 2015 Diurnality as an energy-saving strategy: energetic consequences of temporal niche switching in small mammals. J. Exp. Biol. **218**, 2585–2593. (10.1242/jeb.119354) 26290592

[48] Pembury Smith MQR, Ruxton GD. 2021 Size-dependent predation risk in cryptic prey. J. Ethol. **39**, 191–198. (10.1007/s10164-021-00691-5)

[49] Remmel T, Tammaru T. 2009 Size-dependent predation risk in tree-feeding insects with different colouration strategies: a field experiment. J. Anim. Ecol. **78**, 973–980. (10.1111/j.1365-2656.2009.01566.x) 19493131

[50] Anderson SR, Wiens JJ. 2017 Out of the dark: 350 million years of conservatism and evolution in diel activity patterns in vertebrates. Evolution **71**, 1944–1959. (10.1111/evo.13284) 28636789

[51] Roll U, Dayan T, Kronfeld-Schor N. 2006 On the role of phylogeny in determining activity patterns of rodents. Evol. Ecol. **20**, 479–490. (10.1007/s10682-006-0015-y)

[52] Maor R, Dayan T, Ferguson-Gow H, Jones KE. 2017 Temporal niche expansion in mammals from a nocturnal ancestor after dinosaur extinction. Nat. Ecol. Evol. **1**, 1889–1895. (10.1038/s41559-017-0366-5) 29109469

[53] Odell TM, Mastro VC. 1980 Crepuscular activity of gypsy moth adults. Environ. Entomol. **9**, 613–617. (10.1093/ee/9.5.613)

[54] Borges RM, Somanathan H, Kelber A. 2016 Patterns and processes in nocturnal and crepuscular pollination services. Q. Rev. Biol. **91**, 389–418. (10.1086/689481) 29562117

[55] Endler JA. 1978 A predator’s view of animal color patterns. In Evolutionary biology (eds MK Hecht, WC Steere, B Wallace), pp. 319–364. New York, NY: Springer. (10.1007/978-1-4615-6956-5_5)

[56] Loeffler-Henry K, Kang C, Sherratt TN. 2023 Evolutionary transitions from camouflage to aposematism: hidden signals play a pivotal role. Science **379**, 1136–1140. (10.1126/science.ade5156) 36927015

[57] Mappes J, Kokko H, Ojala K, Lindström L. 2014 Seasonal changes in predator community switch the direction of selection for prey defences. Nat. Commun. **5**, 5016. (10.1038/ncomms6016) 25247589 PMC4199109

[58] Håstad O, Ödeen A. 2008 Different ranking of avian colors predicted by modeling of retinal function in humans and birds. Am. Nat. **171**, 831–838. (10.1086/587529) 18429674

[59] Seddon N, Tobias JA, Eaton M, Ödeen A. 2010 Human vision can provide a valid proxy for avian perception of sexual dichromatism. Auk **127**, 283–292. (10.1525/auk.2009.09070)

[60] Roberts DA, Ustin SL, Ogunjemiyo S, Greenberg J, Dobrowski SZ, Chen J, Hinckley TM. 2004 Spectral and structural measures of northwest forest vegetation at leaf to landscape scales. Ecosystems **7**, 545–562. (10.1007/s10021-004-0144-5)

[61] Eguchi E, Meyer-Rochow V. 1983 Ultraviolet photography of forty-three species of Lepidoptera representing ten families. Annot. Zool. Jpn. **56**, 10–18. (10.34434/za001858)

[62] Rund SSC, Hou TY, Ward SM, Collins FH, Duffield GE. 2011 Genome-wide profiling of diel and circadian gene expression in the malaria vector Anopheles gambiae. Proc. Natl Acad. Sci. USA **108**, E421–30. (10.1073/pnas.1100584108) 21715657 PMC3156198

[63] Zhang R, Lahens NF, Ballance HI, Hughes ME, Hogenesch JB. 2014 A circadian gene expression atlas in mammals: implications for biology and medicine. Proc. Natl Acad. Sci. USA **111**, 16219–16224. (10.1073/pnas.1408886111) 25349387 PMC4234565

[64] Warrant E, Somanathan H. 2022 Colour vision in nocturnal insects. Phil. Trans. R. Soc. B **377**, 20210285. (10.1098/rstb.2021.0285) 36058247 PMC9441242

[65] Yoo S, Kim Y, Lim W, Rim D, Loeffer-Henry K, Sherratt TN *et al*. 2025 Supplementary material from: Coevolutionary patterns between coloration and diel activity in moths. FigShare (10.6084/m9.figshare.c.7881949) PMC12308327 40740705

